# Obstructive hydrocephalus masquerading as impending eclampsia in a 37-week pregnant woman: a case report

**DOI:** 10.3389/fmed.2026.1829568

**Published:** 2026-04-24

**Authors:** Jie Huang, Jianhao Sun, Liangsen Teng, Zhenzhen Wu, Shiyu Li, Jiaqi Chen, Haiyan Liu, Shumei Tuo, Lianying Li, Qing Liu, Xinjuan Jiao, Xiaohua Ding

**Affiliations:** 1 School of Nursing, Gansu University of Chinese Medicine, Lanzhou, China; 2 Nursing Department, Gansu Provincial Maternity and Child-care Hospital (Gansu Provincial Central Hospital), Lanzhou, China; 3 Institute of Translational Medicine, Medical College, Yangzhou University, Yangzhou, China; 4 Clinical Medical College of Youjiang Medical University for Nationalities, Baise, China; 5 Qingyang Second People’s Hospital, Qingyang, China

**Keywords:** neuroimaging, obstructive hydrocephalus, persistent headache, pregnancy, severe preeclampsia

## Abstract

**Background:**

In preeclampsia, the neurologic symptoms, especially a lingering headache, can sometimes mimic more severe intracranial conditions, pushing doctors to quickly consider early delivery. Obstructive hydrocephalus during pregnancy is an uncommon but tricky diagnosis, one that could easily be missed when hypertension and proteinuria are already present, potentially delaying the identification of a neurosurgical emergency.

**Case presentation:**

A 25-year-old first-time mother, 37 weeks and 1 day pregnant, presented with a month-long history of progressively worsening bilateral lower extremity edema and a two-day episode of intense occipital and bitemporal headache. Her blood pressure was elevated at 161/120 mmHg, accompanied by proteinuria (2+), hypoalbuminemia, hyperuricemia, and signs of potential fetal growth restriction, all meeting the criteria for severe preeclampsia. Despite treatment with antihypertensive medications and supportive care, the headache did not subside, prompting an emergency cesarean section. A healthy female infant was delivered, weighing 1960 g, with Apgar scores of 9 at 1 min and 10 at 5 min. Post-surgery, the ongoing neurological concerns led to imaging studies. A CT scan and MRI of the brain revealed significant ventriculomegaly in the supratentorial region, with marked dilation of the lateral and third ventricles, while the fourth ventricle remained unaffected—indicative of obstructive hydrocephalus. Her neurological exam was mostly unremarkable, except for a right-sided Babinski sign. A lumbar puncture showed clear cerebrospinal fluid with an opening pressure of 105 mmH₂O. Neurosurgeons advised close monitoring, and a brief course of mannitol completely alleviated the headache by the fourth postoperative day. She was discharged on day six in good condition.

**Conclusion:**

This case represents a rare instance where obstructive hydrocephalus was diagnosed at term, coexisting with severe preeclampsia, and initially mistaken for impending eclampsia without the usual signs of increased intracranial pressure. Any persistent or unusual headache, particularly when accompanied by focal neurological symptoms, should prompt immediate neuroimaging in pregnant women with hypertension to rule out secondary intracranial causes.

## 1 Introduction

Preeclampsia is a hypertension-related disorder that only occurs during pregnancy, and it can have severe effects on both the mother and fetus. In extreme cases, neurological symptoms such as intense headaches, vision problems, hyperreflexia, and seizures may arise (1). These manifestations can closely mimic those of intracranial pathology, such as hydrocephalus, cerebral venous sinus thrombosis (CVST), intracranial hemorrhage, and posterior reversible encephalopathy syndrome (PRES) (2). Hydrocephalus, though, is rare during pregnancy (3). Its etiologies mainly include shunt dysfunction in patients with a history of hydrocephalus, newly developed obstructive lesions during pregnancy, as well as cerebrospinal fluid circulation or absorption disorders secondary to hemorrhage, infection, or tumors (4–6). When its symptoms are mistaken for those of preeclampsia, the diagnosis is often delayed. In this report, we present the case of a patient in the later stages of pregnancy who exhibited signs pointing to severe preeclampsia. However, after delivery, neuroimaging revealed ventricular enlargement, which aligned with a diagnosis of obstructive hydrocephalus. To the best of our knowledge, cases of term obstructive hydrocephalus complicated by severe preeclampsia that primarily manifest as persistent headache without typical signs of intracranial hypertension are extremely rare. The uniqueness of this case lies in the improvement of symptoms following postpartum conservative management under neurosurgical observation. The patient granted her written informed consent for the anonymous publication of her case details.

## 2 Case presentation

A 25-year-old first-time mother (G1P0) at 37 weeks and 1 day of pregnancy came in with a month-long history of gradually worsening bilateral leg swelling and a persistent headache that had been bothering her for 2 days. She mentioned a cold-like upper respiratory illness prior to the headache but denied any nausea or vomiting. Denial of visual blurring, scotomas, photophobia, and right upper quadrant pain. Her medical history was unremarkable—no chronic hypertension, diabetes, kidney issues, autoimmune conditions, trauma, allergies, or relevant family health concerns. Upon admission, her vital signs were stable: temperature of 36.8 °C, pulse at 80 bpm, respiratory rate of 20 breaths per minute, and blood pressure at 161/120 mmHg. Physical exam showed bilateral pitting edema (++), while her cardiopulmonary findings were normal. On the obstetric exam, the fetus was cephalic, positioned in the left occiput anterior, with a regular fetal heart rate of 145 bpm. The membranes were intact, with a small amount of light red discharge noted.

Laboratory results revealed proteinuria (2+), hypoalbuminemia (32.1 g/L), and hyperuricemia (422 μmol/L). Mildly elevated LDH (315 U/L), normal ALT (6.3 U/L) and AST (12.8 U/L), a normal platelet count (191 × 10^9^/L), and a normal creatinine level (45 μmol/L) were found. Coagulation tests showed raised fibrinogen (4.13 g/L) and D-dimer (1.26 mg/L), while PT and APTT remained normal. An obstetric ultrasound suggested possible fetal growth restriction (estimated weight ~2,200 g), with a high uterine artery pulsatility index and an early diastolic notch on the left side. The Doppler indices for the umbilical artery and middle cerebral artery were within normal limits. Fundoscopic examination revealed no papilledema. Severe preeclampsia was diagnosed.

The initial treatment regimen included oral labetalol 100 mg three times daily, combined with magnesium sulfate for seizure prophylaxis and supportive care. However, the patient’s blood pressure fluctuated between 120–150/80–100 mmHg. However, the headache persisted, leading to an emergency cesarean section at term due to severe preeclampsia and notable neurological symptoms. A female neonate was delivered, weighing 1960 g with Apgar scores of 9 at 1 min and 10 at 5 min.

**Figure 1:**
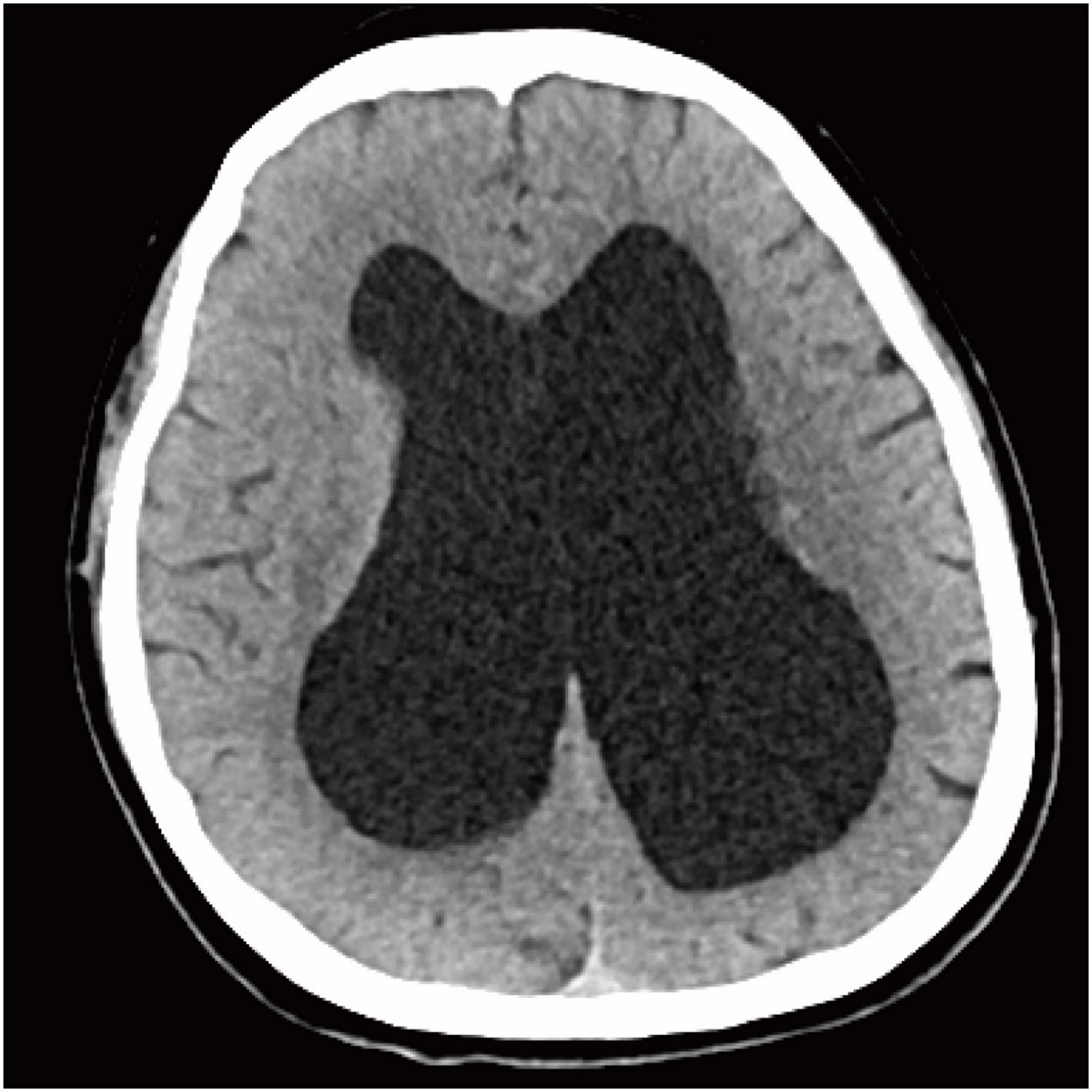
Representative axial head CT demonstrating hydrocephalus. Axial CT image (brain window) shows marked, symmetric dilatation of the bilateral lateral ventricles, with ballooning of the frontal horns and prominent enlargement of the ventricular bodies/occipital horns, accompanied by thinning of the surrounding cerebral mantle, consistent with severe ventriculomegaly/hydrocephalus.

**Figure 2:**
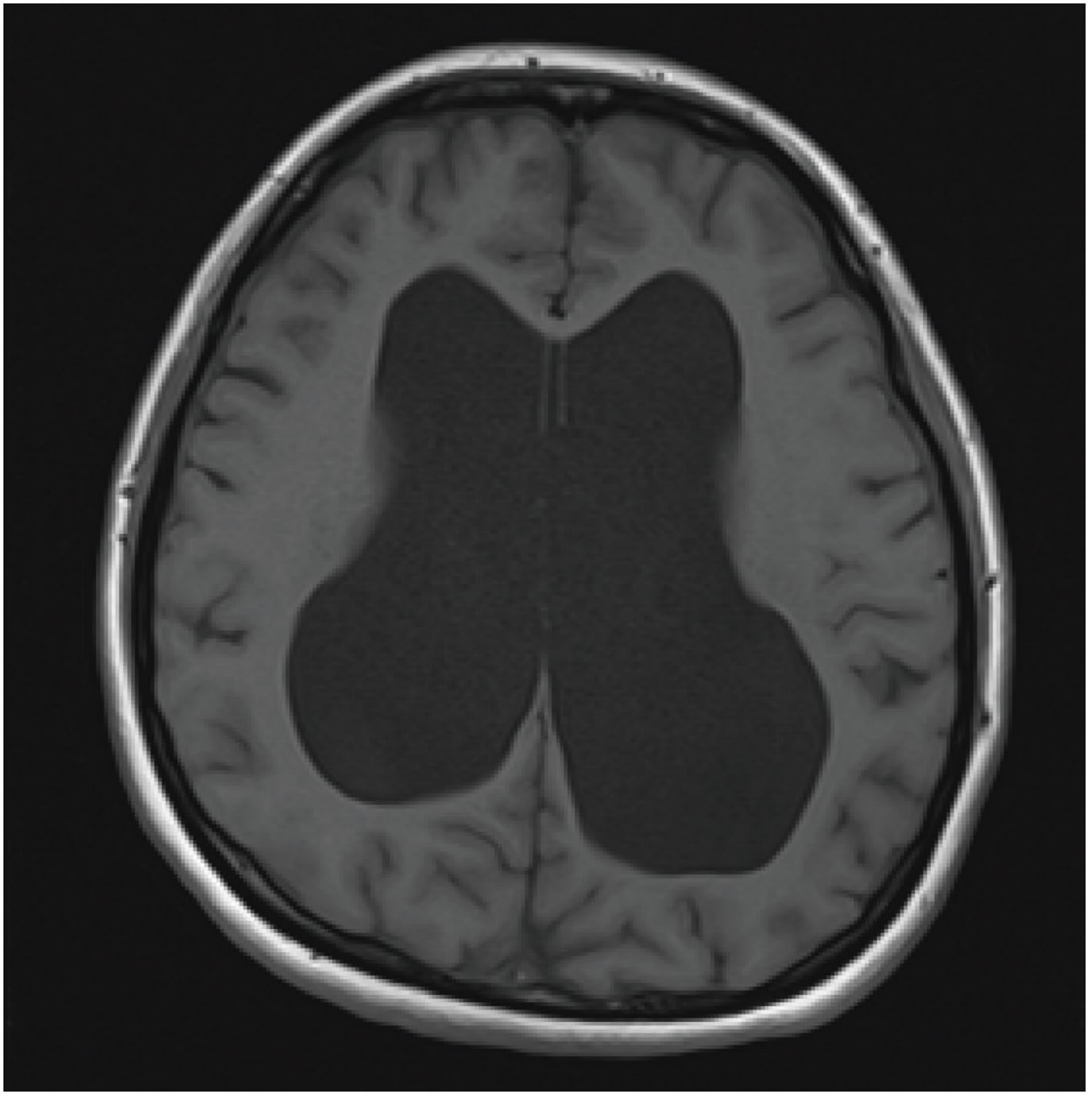
Representative axial brain MRI demonstrating hydrocephalus. Axial T1-weighted MRI image shows marked, symmetric dilatation of the bilateral lateral ventricles with thinning of the surrounding cerebral mantle, consistent with severe ventriculomegaly/hydrocephalus.

Post-surgery, she was moved to the intensive care unit for further assessment. As shown in Figure 1, a CT scan of the head revealed significant enlargement of the lateral ventricles (left ~3.7 cm, right ~4.6 cm) along with patchy periventricular hypodensity and dilation of the third ventricle (~3.4 cm). The fourth ventricle and cerebral aqueduct appeared unaffected. Later, an MRI (Figure 2) confirmed the diagnosis of supratentorial ventriculomegaly (max width: left lateral ventricle ~5.1 cm, right ~4.4 cm, and third ventricle ~3.0 cm), with no abnormal cerebellar signals detected. Neurosurgical consultation with neurological examination revealed that the patient was alert and oriented, with normal muscle strength in all four limbs (5/5). However, a positive right Babinski sign was noted. Lumbar puncture was subsequently performed, showing an opening cerebrospinal fluid (CSF) pressure of 105 mmH₂O. The CSF flowed slowly and appeared clear and transparent. The neurosurgeon recommended close monitoring, with no immediate need for cerebrospinal fluid shunting. Osmotherapy with mannitol was administered and discontinued on postoperative day 4, after which her headache resolved completely, without any rebound symptoms. The patient made a full recovery and was discharged on postoperative day 6. Due to personal reasons of the patient, follow-up neuroimaging could not be completed; a timeline of key clinical events is outlined in Table 1.

**Table 1:** Timeline of clinical course.

Time point	Events
~1 month before admission	Progressive bilateral lower-limb pitting edema
2 days before admission	Persistent occipital/bitemporal headache; preceding cold-like symptoms
Admission (37 + 1 weeks)	BP 161/120 mmHg; proteinuria 2+; hyperuricemia; hypoalbuminemia; suspected FGR
After initial stabilization	BP 120–150/80–100 mmHg; headache persisted
Same day	Emergency cesarean delivery; female neonate 1960 g; Apgar 9/10
Postoperative ICU	CT and MRI: marked lateral and third ventriculomegaly; fourth ventricle not enlarged
Postoperative evaluation	LP: clear CSF; opening pressure 105 mmH₂O; neurosurgery advised observation
Postoperative day 4	Mannitol stopped; headache resolved without rebound
Postoperative day 6	Discharged; no follow-up imaging

## 3 Discussion

This case highlights a diagnostic challenge at the intersection of obstetrics and neurocritical care. A term patient, diagnosed with preeclampsia with severe features, presented with a prominent neurologic symptom—severe headache—that initially suggested an impending eclamptic crisis. However, postpartum imaging revealed significant supratentorial ventriculomegaly, which pointed to obstructive hydrocephalus instead. While modern definitions include neurologic symptoms as a marker of severity, pushing for quicker delivery, headache remains a nonspecific symptom that can overlap with other intracranial diseases (7–9). The key takeaway from this case is that obstructive hydrocephalus can develop late in pregnancy, presenting without the classic signs of raised intracranial pressure—no vomiting, visual changes, altered consciousness, or papilledema—and still mimic the neurologic distress that typically triggers immediate obstetric intervention.

At presentation, several features supported a diagnosis of severe preeclampsia, justifying urgent obstetric action: notably, severe hypertension, proteinuria, biochemical abnormalities (like hyperuricemia and hypoalbuminemia), and Doppler findings suggesting placental insufficiency. At 37 weeks, the recommendation is to deliver, as this is standard for cases with severe features (7, 8). But the persistence of a debilitating occipital-bitemporal headache, despite some improvement in blood pressure, combined with the appearance of a focal neurologic sign (a unilateral Babinski response), created a mismatch that called for further investigation. The turning point in diagnosis came with postpartum imaging. A CT scan and MRI showed disproportionate dilation of the lateral and third ventricles, along with periventricular hypodensity, but no signs of primary hypertensive encephalopathy. This pointed to a structural issue, likely obstructive hydrocephalus. Studies support that in high-risk cases like this, neuroimaging can often provide critical, secondary diagnoses—particularly when neurologic signs are present—emphasizing a low threshold for imaging in such pregnancies (10–12).

In the peripartum setting, the clinically consequential differential diagnosis for severe headache in a hypertensive pregnancy includes PRES, CVST, reversible cerebral vasoconstriction syndrome (RCVS), and intracranial hemorrhage or ischemic stroke (2, 13, 14). In this patient, the absence of seizures, visual disturbance, or encephalopathy, and the lack of MRI findings typical for posterior vasogenic edema, made PRES less supported, although blood pressure status alone cannot exclude it (9). CVST was not favored by the clinical pattern (no seizures or impaired consciousness) and there was no reported imaging evidence of venous infarction/hemorrhage; however, CVST remains a “must-not-miss” diagnosis in pregnancy/postpartum given hypercoagulability and variable presentations (14). RCVS most often presents with thunderclap headache and angiographic vasoconstriction, neither of which was described here (13). Large intracranial hemorrhage or territorial infarction was not suggested by preserved mental status and the absence of focal parenchymal lesions on MRI.

Instead, the imaging findings pointed clearly to obstructive hydrocephalus. The CT scan showed prominent dilation of the lateral and third ventricles, with sparing of the fourth, suggesting non-communicating hydrocephalus. The periventricular hypodensity observed was consistent with transependymal CSF movement, a sign of raised intraventricular pressure typical of obstructive hydrocephalus (15). The MRI reinforced this, showing no abnormalities in the gray-white matter differentiation, no signs of restricted diffusion, and no focal signal abnormalities. Although a lumbar puncture revealed a low-normal opening pressure, this did not rule out the diagnosis. CSF pressure readings are sensitive to the timing of symptom onset, technical factors, and even recent osmotherapy. The variation in CSF pressure readings during clinical practice is well-documented (16). In cases of obstructive hydrocephalus, lumbar CSF pressure may not fully reflect the pressure dynamics inside the ventricles after clinical stabilization.

The mechanism by which hydrocephalus declared itself late in gestation remains uncertain because no antenatal neuroimaging was performed and post-discharge imaging was unavailable. Nonetheless, physiology-based pathways can plausibly align the timing and the symptom overlap with preeclampsia. Normal pregnancy involves plasma volume expansion, reduced oncotic pressure, and changes in venous capacitance that can alter cerebral venous return and intracranial compliance (17, 18). Preeclampsia adds endothelial dysfunction, blood–brain barrier perturbation, and altered cerebrovascular autoregulation, all of which can lower the threshold for neurologic symptoms and amplify headache—even when the primary driver is structural rather than purely hypertensive (9, 19). In a patient with an unrecognized CSF outflow restriction, these hemodynamic and osmotic shifts could precipitate decompensation without a mass lesion evident on conventional MRI sequences. While this is hypothesis-generating rather than causal proof, it provides a biologically plausible explanation for why the headache persisted despite obstetric stabilization and why improvement occurred with postpartum normalization and short-course osmotherapy.

Management in this case highlights the need for careful balancing between managing the maternal-fetal risks of severe preeclampsia and recognizing neurologic emergencies. Delivery at 37 weeks aligns with guidelines for managing severe preeclampsia (7, 8). Postpartum, prompt imaging redefined the neurologic diagnosis. The neurosurgical recommendation to observe rather than perform immediate CSF diversion was based on the stable neurologic exam and the resolution of symptoms with conservative treatment. Still, the broader literature suggests that obstructive hydrocephalus, if symptomatic, can progress rapidly, necessitating definitive CSF diversion, even during pregnancy when necessary. The lack of headache recurrence on follow-up is reassuring, but the absence of further imaging means the full resolution or stability of the hydrocephalus cannot be confirmed, leaving some uncertainty (20–22).

Prior case reports highlight how obstructive hydrocephalus, caused by structural intracranial conditions, can resemble eclampsia or severe preeclampsia and become catastrophic when missed. For instance, a colloid cyst in the third ventricle was initially mistaken for pseudoeclampsia and only identified after the patient’s condition worsened postpartum, resulting in irreversible damage (23). On the flip side, hydrocephalus has also been reported as a complication of intracranial hemorrhage in severe preeclampsia/HELLP with eclampsia, requiring surgical intervention like ventricular drainage (24). Compared to these cases, this report provides two important insights: (1) obstructive hydrocephalus can coexist with severe preeclampsia, and (2) ventriculomegaly can be clinically significant even without typical signs like vomiting or papilledema, and it may improve with conservative care if neurologic status remains stable. These points suggest that the neurologic symptoms in preeclampsia should prompt both obstetric management and a thorough neurologic workup, not just a singular diagnosis.

The practical takeaway is not that every preeclamptic patient with a headache needs an immediate scan, but rather that some patients should be quickly assessed through neuroimaging. When the likelihood of a secondary intracranial issue is higher, the consequences of missing such a diagnosis are too significant to ignore (10, 12). High-risk indicators include: (1) severe headache that does not improve with blood pressure control, (2) focal neurologic signs (even mild ones), (3) new or rapidly worsening headaches, and (4) persistent or recurrent headaches postpartum. In these cases, a head CT or MRI should be done promptly. It’s also important to note that concerns about radiation should not prevent necessary imaging in cases where maternal neurologic emergencies are suspected (25, 26). A multidisciplinary approach involving obstetrics, neurology, neurosurgery, and radiology can help optimize delivery timing, imaging, and the management of potential CSF diversion while ensuring maternal safety.

This report has limitations, including the absence of antenatal neuroimaging, which means we cannot confirm whether hydrocephalus was pre-existing or developed late in pregnancy. We also lacked post-discharge imaging to track radiologic changes. Still, this case underscores that obstructive hydrocephalus can coexist with severe preeclampsia and present primarily as a persistent headache without the typical signs of increased intracranial pressure. Future studies, including prospective multicenter case series, are needed to better define imaging criteria for peripartum headaches and to evaluate the best approach to managing obstructive hydrocephalus—whether through conservative methods or definitive CSF diversion.

## 4 Conclusion

In summary, in the context of severe preeclampsia, if headache persists, exhibits an atypical character, or is accompanied by focal neurological signs, neuroimaging evaluation and multidisciplinary collaboration should be promptly initiated to minimize misdiagnosis and treatment delay.

## Data Availability

The original contributions presented in the study are included in the article/supplementary material, further inquiries can be directed to the corresponding authors.
